# Current Scenario of Postcholecystectomy Bile Leak and Bile Duct Injury at a Tertiary Care Referral Centre of Nepal

**DOI:** 10.1155/2020/4382307

**Published:** 2020-04-21

**Authors:** Narendra Pandit, Tek Narayan Yadav, Laligen Awale, Kunal Bikram Deo, Yogesh Dhakal, Shailesh Adhikary

**Affiliations:** ^1^Surgical Gastroenterology Division, Department of Surgery, B. P. Koirala Institute of Health Sciences (BPKIHS), Dharan, Nepal; ^2^Department of Anaesthesiology and Critical Care, B. P. Koirala Institute of Health Sciences (BPKIHS), Dharan, Nepal

## Abstract

**Objective:**

With the adoption of safe cholecystectomy principles at an academic institute, the risk of major bile duct injury has decreased. This study aims at evaluating the present status of bile duct injury, compared to the study published in 2013 by index centre.

**Methods:**

This is a retrospective review of a prospectively maintained database of bile leak and bile duct injury from 2014 to 2019. Patients who completed postcholecystectomy bile leak or bile duct injury treatment and were on regular follow-up were included.

**Results:**

Eighteen patients (0.78%) among 2,300 consecutive cholecystectomies presented with bile duct injury, including 8 (0.35%) major bile duct injuries and 10 (0.43%) bile leaks compared to major bile duct injury rate of 0.68% (92/11,345 cholecystectomies) between 2001 and 2010. Injuries were classified as Strasberg's type A (52.9%), type D (5.9%), and type E (41.1%). Eight patients (47%) of bile leak were managed conservatively with drains, while two required laparotomy and lavage. The mean time for spontaneous closure of bile leak was 11 days. Intraoperative repair was done in three cases: Roux en Y hepaticojejunostomy in 2 and end-to-end repair over T-tube in 1 for sharp transection of the duct. Delayed repair (Roux-en-Y hepaticojejunostomy) was done in five patients. The median postcholecystectomy hospital stay was 8 days, with no mortality. There was no restricture at a median follow-up of 13 months.

**Conclusion:**

With the adoption of a safe culture of cholecystectomy, the major bile duct injury rate has decreased currently. Repair of bile duct injury by experienced hepatobiliary surgeon results in excellent outcome.

## 1. Introduction

Cholecystectomy is the most common operation performed worldwide. It is commonly performed by laparoscopic or by open method in case of difficult anatomy or pathology [1]. Both the procedures are associated with the risk of bile duct injury, with the risk being 0.4–0.6% and 0.2-0.3% for laparoscopic and open cholecystectomy, respectively [2]. The bile duct injury significantly increases the healthcare cost, morbidity, and mortality and decreases survival [3, 4]. However, the data of risk stratification of bile duct injury are a decade old. Nowadays, with the adoption of principles of safe cholecystectomy and extravigilance at the academic institute, the risk of major bile duct injury has somehow decreased [5]. On the contrary, the incidence of bile leak following laparoscopic cholecystectomy has increased in recent days [4, 6]. In 2013, the rate of bile duct injury, management, and its outcome (2001–2010) was studied from our institute [7]. In the present study, we aimed at studying the present status of the rate of bile leak and (major) bile duct injury, its management, and outcome, a decade later at our centre.

## 2. Materials and Methods

This series represents a retrospective review of a prospectively maintained database of all patients who developed bile leak and bile duct injury from April 2014 to May 2019 at our tertiary referral centre. The institute is a 750-bedded, academic centre with a separate HPB unit. Inclusion criteria were patients who completed the treatment for bile leak and bile duct injury due to laparoscopic/open cholecystectomy and were on regular follow-up. Those patients who refused for intervention, surgery, incomplete treatment, lost to follow-up, and cholecystectomy combined with other abdominal procedures were excluded (*n* = 4). The study was approved by the Institutional Ethical Board.

Bile leak was defined as leak from the cystic duct stump or the aberrant bile ducts with maintained continuity of the extrahepatic duct and appearance of bile from the surgical or percutaneous drainage. It was later confirmed by normal ultrasound, liver function tests, or magnetic resonance cholangiography (MRCP). Similarly, (major) bile duct injury was defined as all transaction, segment loss, or stenosis of the extrahepatic bile duct or major segmental ducts requiring hepaticojejunostomy or end-to-end bile duct anastomosis, or undergoing more than 1 endoscopic retrograde cholangiopancreatography (ERCP) within a year of cholecystectomy. Imaging investigations performed to diagnose the bile duct injury and leak were ultrasound, contrast computed tomography (CT), and MRCP depending on the presentation and severity of the injury.

Medical records were examined individually to extract data on demographics, type of cholecystectomy, indications for cholecystectomy, number of cases referred from other centre vs. injuries at index hospital, mode of presentation, timing of detection of injury, type of injury as per Strasberg's classification [2], and type of intervention (conservative vs. surgery). Type of surgery, timing of repair, postoperative morbidity, mortality, total length of hospital stay, and follow-up were also recorded. Whenever possible, the data were presented as a standard tabular reporting format specific for biliary injury as proposed by Cho et al. [8].

Statistical analysis was performed with SPSS v 17.0 software for the descriptive statistical analysis by calculating mean, median, standard deviation, and percentage where appropriate. To see the trend of bile duct injury at our centre, compared to the study published in 2013, a *Z*-test for two sample proportions was used. A *P* value <0.05 was considered to be significant.

## 3. Results

Eighteen patients presented with bile duct injury among 2,300 consecutive cholecystectomies (0.78%), including 8 (0.35%) major bile duct injuries and 10 (0.43%) bile leaks. After excluding five injuries that were referred from other centre, the current incidence of bile duct injury and major injury was 0.56% (13/2,300 cholecystectomies) and 0.21% (5/2,300 cholecystectomies) respectively. These injuries were seen in 11 females and 7 males, with a mean age of 40 years. Cholecystectomy was performed laparoscopically (4-port) in 15 (83.3%) and by open in 3 (16.7%) patients. Four (22.2%) injuries were detected intraoperatively, while the remaining 14 (77.8%) were detected in the postoperative period.

**Figure 1:**
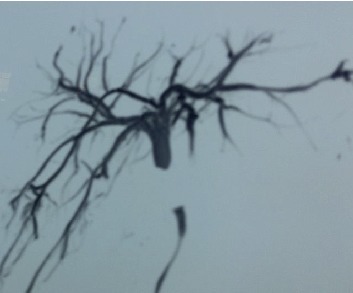
Magnetic resonance cholangiopancreatography (MRCP) showing Strasberg's type E2 injury following open cholecystectomy.

As per Strasberg's classification system, the injuries were classified as type A in 9(50%), type D in 1(5.5%), and type E in 8(44.5%) patients (Figure 1). Among E class, E1-1, E2-1, and E3 in 2 patients were seen. Five patients sustained type E injury at index hospital. There were no associated vascular injuries. Eight patients (44.4%) were managed conservatively (Tables 1 and 2). Among them, 2 had indwelling surgical drains (one difficult cholecystectomy and the other following intraoperative closure of iatrogenic common hepatic duct rent) following laparoscopic cholecystectomy, which presented with controlled external biliary fistula, low output (<200 ml), and ceased spontaneously within a week. The remaining 6 patients presented with bilioma with sepsis, required image-guided percutaneous catheter drainage (PCD) (1 PCD-2, 2 PCD-1, and 3 PCD-1), antibiotics, source control, and achieving controlled external biliary fistula. The leak subsided spontaneously at a mean time of 11 days (range: 4–34 days). None required endoscopic intervention (stenting/sphincterotomy), as the fistula volume was low-output, decreasing trend, improving the general condition of the patients and an unavailability of service at our centre at the time of writing of this paper.

**Figure 2:**
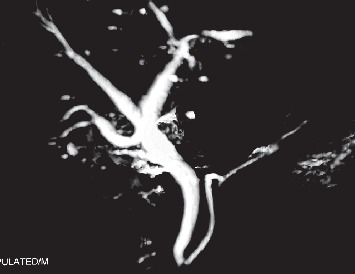
Follow-up magnetic resonance cholangiopancreatography (MRCP) showing normal extrahepatic bile duct with bilioenteric continuity, following sealed cystic duct stump leak (Strasberg's type A injury).

Ten (55.5%) among 18 patients required surgical intervention. There were seven Roux-en-Y-hepaticojejunostomy (Hepp-Couinaud approach) by the experienced hepatobiliary surgeon, five delayed and two performed intraoperatively. One patient required end-to-end common bile duct repair over the T-tube (detected intraoperatively) for complete transection without segment loss during open cholecystectomy. The remaining 2 patients required emergency laparotomy, peritoneal lavage, and drain placement for peritonitis due to the class A Strasberg injury (confirmed by postoperative MRCP) (Figure 2). Postoperatively, two patients developed superficial surgical site infections (SSIs). There was no mortality in our series of patients (Table 2). The median length of hospital stay postcholecystectomy was 8 days (range: 5–28 days). There was no restricture at a median follow-up of 13 months (range: 8–36 months), as confirmed by history and clinical examination, liver function tests, and ultrasonography (Table 3). When the trend of injuries was compared, there was a significant decrease in incidence (0.21% vs. 0.68%; *P* = 0.007) of major bile duct injury at index hospital (Table 4).

## 4. Discussion

Bile leak and major bile duct injury are the most feared complications after open and laparoscopic cholecystectomy. It significantly increases the morbidity, mortality, and costs of hospital stay and decreases the quality of life [5, 9]. Furthermore, it is associated with litigations. Despite increasing experience and familiarity, incidence of common bile duct injury following laparoscopic and open cholecystectomy still continues to be 0.4% to 0.6% and 0.2% to 0.3%, respectively [2, 10]. On the contrary, the rate of bile leak has increased in recent decades (1.5% to 3%) [4, 11]. In the present study, the rate of bile leak and major duct injury was 0.43% and 0.35%, respectively, which is in line with the published international standard [5].

In a study published by Gupta et al. [7], from our institute (study period: 2001 to 2010), there was a high rate of major bile duct injury (0.68%). The major bile duct injury over the decade, where the laparoscopic approach was flourishing, was seen in 92 patients (among 11,345 cholecystectomies), with 83 patients requiring bilioenteric anastomosis. The mortality rate from the injury was 3.3%. Certainly, a decade later at the same institute, the rate of major bile duct injury (0.21% vs. 0.68%; *P*=0.007) at index hospital and mortality from it have decreased significantly. This can be attributed to the “culture of safe cholecystectomy,” by adopting critical view of safety, Rouviere's sulcus as a landmark for initiation of dissection, bail-out options or early conversion in difficult cholecystectomy, time-out and in vicinity “colleaguography” before clipping, and extravigilance in an academic centre for the risk and consequences of bile duct injury [4, 12–14].

Similarly, the rate of bile leak from the cystic duct stump or the aberrant/duct of Luschka (Strasberg class A injury) has increased by laparoscopic approach. The bile leak is equally dangerous, if presented late with sepsis or the diagnosis is delayed [15]. In a study by Viste et al. [1], the rate of bile leak was 0.9%, all attributed from the cystic duct or assumed ducts from liver bed, which compromised 52% of total bile duct injury. One out of four deaths was from peritonitis due to the leak from the cystic duct stump. In our study, nine patients had bile leak from the assumed cystic duct or duct of Luschka and one from common hepatic duct rent closure leak. Among them, two required laparotomy and lavage for peritonitis, while the remaining were managed conservatively with drainage of bilioma. All developed controlled external biliary fistula, which closed spontaneously, with a mean fistula closure time of 11 days. None underwent ERCP, sphincterotomy, or stenting, which nowadays is the treatment modality of choice, as the facilities were not available at our centre [16]. Moreover, due to the logistic reason (financial constraints), lack of health insurance, and geographic status of the country, patient refused to move to other higher centre (700 km) for ERCP endoscopic intervention. There is no debate that early ERCP endoscopic intervention is safe, effective, and considered the first line of therapy in bile leak. It improves the clinical outcome, decreases bilioma formation, decreases the rate of laparotomy and number of percutaneous interventions, removes the missed common bile duct stones as a cause for leak, and decreases time to fistula closure and even deaths [15, 16]. In the present study, fortunately, other than the increased number of percutaneous interventions and increased time to fistula closure, there were no increased laparotomy rates or death due to the conservative management of bile leak.

The important finding of this study is the excellent outcome of patients undergoing repair for major bile duct injury. It has been well described for major bile duct injury that the first repair should be the best repair, not by the primary surgeon, but by the expert hepatobiliary surgeon at the experienced centre [2, 4]. We had a policy of performing delayed repair (>6 weeks), with control of intra-abdominal sepsis and nutritional stabilization of the patients, or an on-table repair if diagnosed intraoperatively. Bilioenteric drainage (Roux-en-Y hepaticojejunostomy in 7 patients) is the preferred treatment option for major duct injury; however, end-to-end common bile duct repair over the T-tube (1 patient) is also the safe option for sharp transaction injuries without segment loss and a nonelectrocautery injury [2, 3]. There was no restricture in those eight patients who required on-table and delayed repair at a median follow-up of 13 months.

The study is limited by its retrospective design, short-time frame, small sample size, lack of state-of-the-art ERCP for the management of bile leak at our centre, and lack of long-term follow-up to detect restricture. Despite this, the study beautifully shows the decreased rates of major bile duct injury at our centre, with good short-term outcomes.

## 5. Conclusion

Bile leak and major duct injury are the most feared complications of cholecystectomy and results in significant morbidity, mortality, and healthcare costs. With the adoption of a safe culture of cholecystectomy, the rate of major bile duct injury has decreased compared to the results of a decade back at our centre. Similarly, the bile leak from the cystic duct/duct of Luschka has been increasingly detected. Repair of major bile duct injury by the nonprimary and experienced hepatobiliary surgeon results in excellent outcome.

## Figures and Tables

**Table 1:** Demographics, details of index operation, presentation, and staging of injury.

Parameters	Total patients, *n* = 18
Age (years), mean (range)	40 (16–55)
Male : female (M : F)	7 : 11

*Place of injury*	
Outside hospital	5 (27.8%)
In hospital	13 (72.2%)

*Indication for cholecystectomy*	
Biliary colic	9 (50%)
Acute cholecystitis	4 (22.2%)
Mucocele	4 (22.2%)
Xanthogranulomatous cholecystitis	1 (5.5%)

*Surgery started as*	
Open	3 (16.7%)
Laparoscopic	15 (83.3%)
Any conversion from laparoscopy to open	0

*Methods of cholecystectomy*	
Dissection of hepatocystic triangle first	13 (72.2%)
Unknown	5 (27.8%)

*Methods of cystic duct identification*	
Critical view of safety	11(61.1%)
Infundibular approach	1(5.5%)
Top-down	1(5.5%)
Unknown	5(27.8%)

*Detection of injury*	
Intraoperative	4 (22.2%)
Postoperative	14 (77.8%)

*Injury occurred during which part of procedure*	
Open	3 (16.7%)
Laparoscopic	15 (83.3%)

*Management of recognized injury*	
End-to-end anastomosis + T-tube	1(5.5%)
Hepaticojejunostomy	2 (11.1%)
Suture closure of rent in common hepatic duct	1 (5.5%)

*Indication for referral (n* *=* *15)*	
Jaundice	5 (29.4%)
Intra-abdominal sepsis	8(35.3%)
Biliary fistula	2 (11.8%)

*Time from index operation to referral for surgical repair*	
Intraoperative	3 (16.7%)
0–3 days	3(16.7%)
4–7 days	3(16.7%)
8 days–6 weeks	5 (27.8%)
6 weeks–3 months	4 (22.2%)

*Staging of injury*	
A	9 (50%)
D	1 (5.5%)
E1	5(27.8%)
E2	1 (5.5%)
E3	2 (11.1%)
Vasculobiliary injury	0
Other organs injured	0

**Table 2:** Preoperative risk assessment, laboratory values, intraoperative events, and outcomes of operative group.

Parameters	Results (*n* = 10)
Type II diabetes mellitus	2 (20%)
Current smoker within 1 year	5 (50%)
Hypertension requiring medication	3 (30%)
Preoperative blood transfusions (red blood cells within 72 hr before surgery)	1 (10%)
Sepsis within 48 hr before surgery	0
Cirrhosis	0
Mean hemoglobin (g/dl)	11.6 ± 1.8
Median total serum bilirubin, mg/dl (range)	2.0 (0.8–18.0)
Albumin (g/dl)	3.9 ± 0.25

*Timing of repair (time from index surgery)*	
<24 hr	3 (30%)
>7 days–<6 weeks	3 (30%)
8 to 12 weeks	4 (40%)

*Procedure done*	
Hepaticojejunostomy	7 (70%)
End-to-end anastomosis + T-tube	1 (10%)
Laparotomy + lavage + drainage	2 (20%)
End-to-side hepaticojejunostomy	2 (20%)
Side-to-side hepaticojejunostomy	5 (50%)
Any form of liver resection	0
Superficial SSI	2 (20%)
Mortality	0

**Table 3:** Results of bile duct reconstruction.

Parameters	
Length of follow-up, median (months)	13 (8–36)
Lost to follow-up	0
Any evidence of restricture or recurrent cholangitis	0
Any postoperative interventions for anastomotic problems	0

**Table 4:** Comparison of the present study with the previous study from our centre.

Parameters	Current study (2014–2019)	Previous study (2001–2010) Gupta et al. [7]
Total cholecystectomies (in-hospital)	2,300	11,345
Mean age, years, (range)	40 (16–55)	46.5 (23–68)
Referred	5 (27.8%)	15 (16.3%)
Index hospital	13 (72.2%)	77 (83.7%)

*Overall incidence*		
Major bile duct injury	8 (0.35%)	
In-hospital major bile duct injury	5 (0.21%)	77 (0.68%) (*Z* = 2.659 and *P*=0.007)
Bile leak	10 (0.43%)	NA
Morbidity	2 (11.1%)	NA
Mortality (30 and 90 days)	0	3 (3.3%)
Follow-up	(*n* = 18)	(*n* = 75)
Median follow-up (mo)	13	31
Restricture	0	3 (4%)
Good outcome	18 (100%)	62 (82%)

## Data Availability

The data used to support the findings of the study are available from the corresponding author upon request.
